# EjNAC3 transcriptionally regulates chilling-induced lignification of loquat fruit via physical interaction with an atypical CAD-like gene

**DOI:** 10.1093/jxb/erx330

**Published:** 2017-09-16

**Authors:** Hang Ge, Jing Zhang, Yi-jin Zhang, Xian Li, Xue-ren Yin, Donald Grierson, Kun-song Chen

**Affiliations:** 1 Zhejiang Provincial Key Laboratory of Horticultural Plant Integrative Biology, Zhejiang University, Zijingang Campus, Hangzhou, PR China; 2 The State Agriculture Ministry Laboratory of Horticultural Plant Growth, Development and Quality Improvement, Zhejiang University, Zijingang Campus, Hangzhou, PR China; 3 Plant and Crop Sciences Division, School of Biosciences, University of Nottingham, Sutton Bonington Campus, Loughborough, UK

**Keywords:** CAD-like, chilling injury, lignin biosynthesis, loquat, NAC, transcriptional regulations

## Abstract

Lignin is an important component of many plant secondary cell walls. In the fruit of loquat (*Eriobotrya japonica*), lignification of cell walls in the fleshy tissue occurs when fruit are subjected to low-temperature storage, which is commonly used to avoid the rapid senescence that occurs at room temperature. In this study, two NAC domain genes, *EjNAC3* and *EjNAC4*, were isolated and shown to be significantly induced at 0 °C, which was concomitant with an increase in the fruit lignification index. Lignification and expression of both *EjNAC3* and *EjNAC4* were inhibited by low-temperature conditioning and by heat treatment. In addition, *EjNAC3* trans-activated the lignin biosynthesis-related *EjCAD-like* promoter, which was measured using a dual-luciferase assay. Further analysis with yeast one-hybrid and electrophoretic mobility shift assays indicated that EjNAC3 could physically bind to the promoter of the *EjCAD*-*like* gene. Thus, *EjNAC3* is a direct regulator of loquat chilling-induced lignification, via regulations of *EjCAD*-*like*.

## Teaser

A novel transcription factor, EjNAC3, is involved in chilling-induced lignification in loquat via direct interaction with a CAD-like gene, which differs from other ‘master-switch’ NAC genes.

## Introduction

Low-temperature storage is routinely applied to extend the post-harvest life of fruit; however, various chilling injury symptoms commonly occur in fruit subjected to inappropriately low temperatures or long-term storage. For example, the skin of pomegranate becomes brown and pitted if stored below 5 °C (Sayyari *et al.*, 2009), while peach fruit stored at 5 °C exhibits more severe symptoms of flesh browning than fruit stored at 0 or 8 °C (Zhang *et al.*, 2011). Unlike other fruit, loquat (*Eriobotrya japonica*), a species of Rosaceae, not only undergoes flesh browning in response to low-temperature storage (0 °C), but also exhibits typical lignification symptoms, such as an increase in lignin content and firmness (Zheng *et al.*, 1999; Cai *et al.*, 2006*d*). Due to the significant impact of lignification on fruit quality, numerous treatments have been developed to manipulate loquat fruit lignification, including low-temperature conditioning (LTC) (Cai *et al.*, 2006*d*), heat treatment (HT) (Xu *et al.*, 2014), salicylic acid treatment (Ding *et al.*, 2002; Cai *et al.*, 2006*b*), methyl jasmonate (MeJA) treatment (Cao *et al.*, 2009), and 1-methylcyclopropene (1-MCP) treatment (Cai *et al.*, 2006*a*). Thus, loquat fruit represents well-researched material on which to investigate the underlying mechanisms controlling fruit-flesh lignification.

Based on research from model plants and woody trees, the biochemical and molecular basis for loquat fruit lignification has been investigated, and it has been shown to be correlated with the activities of enzymes involved in the phenylpropanoid pathway, including L-phenylalanine ammonia lyase (PAL), 4-coumarate:coenzyme A ligase (4CL), and cinnamyl alcohol dehydrogenase (CAD): these enzymes are significantly repressed by treatments with LTC, 1-MCP, acetylsalicylic acid (ASA) (Cai *et al.*, 2006a, b, c, d), and MeJA (Cao *et al.*, 2010), which also reduce lignification.

In model plants and woody trees, transcription factors, especially NAC and MYB, have been widely reported to be involved in lignin biosynthesis and secondary cell wall construction, and a complicated network is formed through interactions at the transcriptional and/or post-translational levels (Kubo *et al.*, 2005; Mitsuda *et al.*, 2005; Zhong *et al.*, 2008; Ohashi-Ito *et al.*, 2010; Yamaguchi *et al.*, 2011). Many MYB-type transcription factors, for example *AtMYB58*, *AtMYB63* (Zhou *et al.*, 2009), *AtMYB85* (Zhong *et al.*, 2008), and *AtBLH6* (Cassan-Wang *et al.*, 2013), are activators of lignin biosynthesis while *AtMYB4*, *AtMYB7*, and *AtMYB32* act as repressors (Preston *et al.*, 2004; Wang and Dixon, 2012), and in some cases the gene promoters they interact with have been identified. For instance, 4CL genes, which function in the upstream part of the phenylpropanoid pathway, were confirmed to be a direct target of both *AtMYB58* and *AtMYB6* (Zhou *et al.*, 2009). In contrast, most NAC-domain proteins operate at the top level of the regulatory network, which means that evidence that these NAC genes physically interact with structural genes has not been found. They are designated as ‘master switches’ of secondary cell wall formation, and include *AtNST1* and *AtNST2*, *AtVND1* to *AtVND7*, and *AtSND1* (Kubo *et al.*, 2005; Mitsuda *et al.*, 2005; Zhong *et al.*, 2006; Bennett *et al.*, 2010), although *AtSND*1 also directly activates expression of *MtF5H*, which encodes ferulate-5-hydroxylase in *Medicago truncatula* by binding to its promoter (Zhao *et al.*, 2010), and all have crucial roles during secondary cell wall formation.

The first transcription factors shown to be involved in loquat fruit-flesh lignification were *EjMYB*1 and *EjMYB2* (Xu *et al.*, 2014). *EjMYB1* was found to be capable of transcriptional activation of a number of lignin biosynthesis-related genes, such as *EjPAL1*, *Ej4CL1*, and *Ej4CL5*, and transient over-expression of *EjMYB1* in tobacco leaves resulted in ectopic accumulation of lignin. *EjMYB2* was characterized as a repressor of loquat fruit lignification, and an AP2/ERF family gene, *EjAP2-1*, indirectly regulated lignification via protein–protein interactions with either *EjMYB1* or *EjMYB2* (Zeng *et al.*, 2015). In addition to MYB transcription factors, *EjNAC1* was also shown to enhance expression during loquat fruit chilling-induced lignification; however, it could not bind directly to promoters of lignin biosynthesis genes (Xu *et al.*, 2015), indicating an indirect mechanism of activation. These reports identified several components of the transcriptional regulatory mechanisms underlying loquat fruit-flesh lignification; however, the relationships between them and the overall control of the pathway are not clear. For instance, all of the known transcription factors, *EjMYB1*, *EjMYB2*, and *EjAP2-1*, have significant regulatory effects on *EjPAL1* and *Ej4CL1* promoters (upstream genes of the phenylpropanoid pathway) but have limited effects on downstream pathway genes.

In the present study, two NAC-domain genes were isolated from loquat fruit. Their transcript levels in response to low-temperature conditioning and heat treatment were characterized, together with their trans-activation functions. In addition, the relationship between these NAC genes and lignification was investigated and potential targets of the NAC genes were screened by dual luciferase assays, and their physical interactions were confirmed using a yeast one-hybrid system and electrophoretic mobility shift assays.

## Materials and methods

### Plant materials and treatments

Fruits of loquat (*Eriobotrya japonica* Lindl., red-fleshed variety ‘Luoyangqing’) were collected in 2011 at Luqiao, Zhejiang province, China. The fruit material and treatments have been described in detail previously by Xu *et al.*, (2014). In brief, uniform fruit were selected and divided into three batches with about 150 fruit in each. One batch was stored at 40 °C for 4 h as the heat treatment (HT) and was then transferred to 0 °C for storage. For the low-temperature conditioning (LTC), fruit were kept at 5 °C for 6 d followed by storage at 0 °C. The third batch of fruit was immediately stored at 0 °C, as a control. Three biological replicates were used for all treatments and samplings.

### Gene isolation and phylogenetic analysis

Differentially expressed unigenes, within the NAC domain, were obtained from previously generated RNA-seq results (Xu *et al.*, 2014). The full coding DNA sequences (CDS) of two genes were amplified with a SMART RACE cDNA amplification kit (Clontech), using the primers listed in Supplementary Table S1 at *JXB* online. These genes were aligned with Arabidopsis NAC genes obtained from the TAIR database (https://www.arabidopsis.org/), using the neighbor-joining (NJ) method with ClustalX (v.1.81). The results of the alignments were visualized using FigTree (v. 1.3.1).

### RNA extraction and cDNA synthesis

Total RNA used to synthesize cDNA was extracted from the fruit flesh according to the protocol described by Shan *et al.*, (2008). Potential genomic DNA contamination was eliminated using a TURBO DNA-free kit (Ambion). The quality and quantity of RNA extracted from the fruit flesh was determined by gel electrophoresis and spectrophotometry (Implen). To synthesize first-strand cDNA, 1 µg DNA-free total RNA was added to a mixture of cDNA synthesis reagents (BioRad).

### Real-time PCR analysis

Real-time PCR was carried out using the CFX96 Real-Time System (Biorad), with SsoFast EvaGreen Supermix (Biorad). The primers were designed using Primer3 (version 4.0.0; http://bioinfo.ut.ee/primer3/) and are listed in Supplementary Table S2. All of the primers were tested to ensure their specificity for unique genes (Yin *et al.*, 2010).

### Dual luciferase assay

Dual luciferase assays (described previously by Yin *et al.*, 2010; Min *et al.*, 2014) were performed to analyse the potential relationships between NAC transcription factors and loquat lignin biosynthesis genes and previously characterized transcription factors involved in loquat lignification. Full-length NAC genes were amplified with primers (listed in Supplementary Table S3) and integrated into the pGreen II 0029 62-SK vector (SK). Promoters of lignin synthesis-related genes from both loquat and Arabidopsis were inserted into the pGreen II 0800-LUC vector (LUC), as described by Xu *et al.*, (2014). Promoters regions of the *EjMYB1*, *EjMYB2*, and *EjAP2-1* transcription factors were constructed into the pGreen II 0800-LUC vector.

All of the recombinant SK and LUC vectors were transfected into *Agrobacterium tumefaciens* GV3101. The glycerol stocks with transfected *Agrobacterium* were grown in lysogeny broth (LB) plates with agar and 50 µg ml^–1^ kanamycin and 25 µg ml^–1^ gentamycin for 2 d and then restreaked onto new LB plates for 1 d. *Agrobacterium* were suspended in infiltration buffer (10 mM MES, 10 mM MgCl_2_, 150 mM acetosyringone, pH 5.6) to an optimal density (OD_600_=0.75), then 1 ml of *Agrobacterium* cultures containing transcription factors were mixed with 100 µl of *Agrobacterium* containing promoters. The mixtures were then injected into tobacco (*Nicotiana tabacum*) leaves with needleless syringes, and 3 d after infiltration the LUC and REN fluorescence intensities were measured using dual luciferase assay reagents (Promega). Five replicates were conducted for each transcription factor and promoter combination.

### Yeast one-hybrid assay

Yeast one-hybrid assays were conducted to test the physical interactions between transcription factors and promoters identified from the dual luciferase assay. Yeast one-hybrid assays were performed using the Matchmaker Gold Yeast One-Hybrid Library Screening System (Clontech, USA). The promoter of *EjCAD-like* was amplified and inserted into the pAbAi vector. Similarly, the pGADT7 vector with full-length *EjNAC3* was constructed (primers are listed in Supplementary Table S4). The recombinant *EjCAD-like*-pAbAi vector was linearized and transformed into the Y1HGold yeast strain. After testing for autoactivation, the Y1HGold[*EjCAD-like*/AbAi] yeast strain was transfected with the EjNAC3-pGADT7 plasmid and grown on SD/–Leu medium containing 0–500 ng ml^–^ of AbA at 30 °C for 3 d. The empty pGADT7 plasmid was transformed as a negative control.

### Electrophoretic mobility shift assay (EMSA)

The intact open reading frame of *EjNAC3* was inserted into pET-32a (Clontech) to generate an EjNAC3-His fusion protein. The reconstructed plasmid was transformed into *Escherichia coli* strain BL21. The transformed cells were treated with 1mM isopropyl β-D-1-thiogalactopyranoside (IPTG) followed by incubation at 16 °C for 20 h. After extraction of the recombined protein, a HisTALON™ Gravity Column (Clontech) was used for protein purification, following the steps described in the official user manual.

A Lightshift Chemiluminescent EMSA kit (Thermo) was used to perform EMSA experiments according to the manufacturer’s instructions. Single-strand oligonucleotides were synthesized and biotinylated by the JINBAIAO company (Shanghai, China). After mixing forward and reverse oligonucleotides together, the mixture was incubated at 95 °C for 5 min and the temperature gradually decreased to 25 °C at the rate of 0.1 °C s^–1^, allowing the formation of double-strand probes. The binding buffer provided with the kit was used together with 5 mM MgCl_2_, 2.5% glycerol, 0.05% NP-40, 50 ng of purified protein 2 nM probe, and 50 ng µl^–1^ of poly(dI-dC) to set reactions. For competition reactions, the cold probe was incubated with the protein for 20 min followed by addition of the biotinylated probe. The solution was analysed using 6% native polyacrylamide gel electrophoresis for 30 min after incubation for 20 min at room temperature. The voltage was set to 120 (constant voltage mode) and 0.5× Tris-borate/EDTA buffer was pre-chilled. The fractionated products were transferred onto nitrocellulose membranes with 0.5× Tris-borate/EDTA at 280 mA (constant electric current mode) for 30 min with cold 0.5× Tris-borate/EDTA buffer (4 °C). Cross-linking was performed under a UV-light for 30 min. The membrane was then incubated in blocking buffer with shaking for 15 min, transferred to conjugate/blocking buffer (16.75 µl stabilized streptavidin-horseradish peroxidase conjugate with 5 ml blocking buffer) and washed four times, each for 5 min with 1× washing buffer. Detection of biotinylated DNA probes was achieved using the ChemiDoc™ MP Imaging System (Bio-Rad). The probes used for EMSAs are listed in Supplementary Table S5.

### Statistical analysis

The statistical significance of differences was determined using Student’s *t*-test. Least-significant differences (LSD) at the 5% level were calculated using DPS7.05 (Zhejiang University, Hangzhou, China). Figures were drawn using Origin 8.0 (Microcal Software Inc., Northampton, MA, USA).

## Results

### Isolation of loquat NAC genes and phylogenetic analysis

**Fig. 1.**
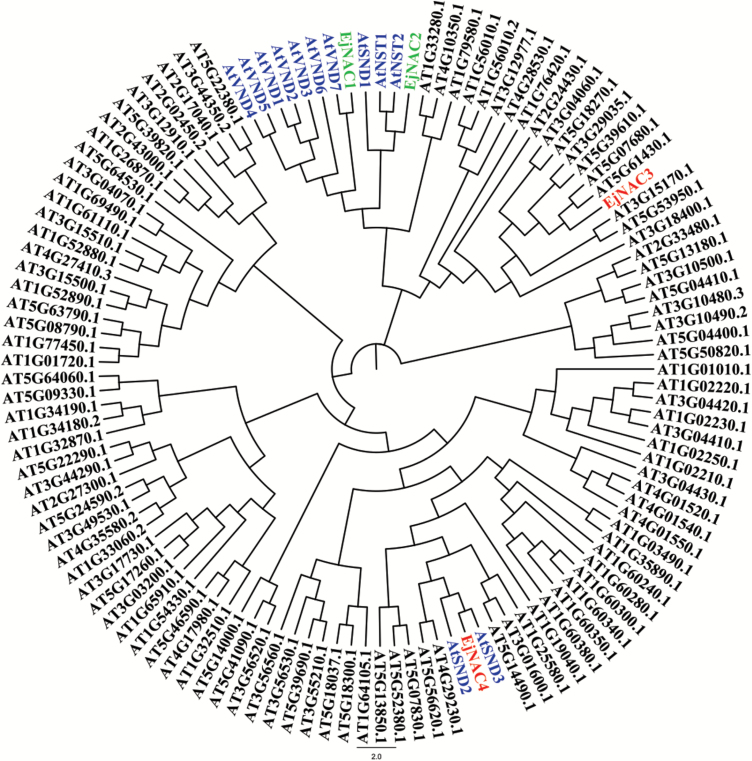
Phylogenetic analyses of loquat NAC genes. Amino acid sequences of Arabidopsis NAC genes were downloaded from TAIR (https://www.arabidopsis.org/). Secondary cell wall-related genes from Arabidopsis are highlighted in blue, and previously reported *EjNAC1* and *EjNAC2* are highlighted in green.

Two loquat NAC genes, *EjNAC3* and *EjNAC4*, were isolated based on unigenes from the loquat RNA-seq database (data not shown). Phylogenetic tree analysis indicated that *EjNAC4* was clustered with lignin-related AtSND2/3, while *EjNAC3* was not clustered with any secondary cell wall-related NAC genes (Fig. 1).

### Expression levels of NAC genes in fruit flesh after HT and LTC treatments

**Fig. 2.**
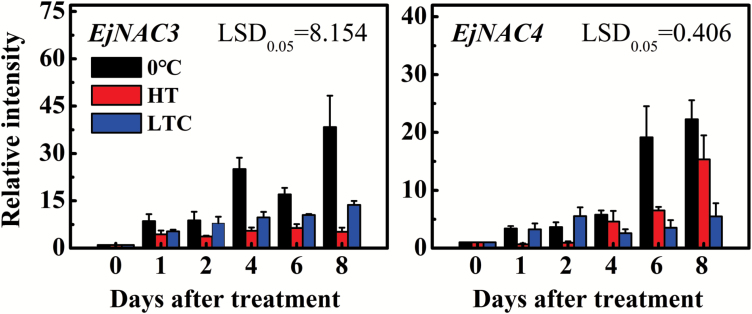
Expression of loquat NAC genes in response to cold (0 °C), heat treatment (HT), and low-temperature conditioning (LTC). Values are presented as relative expression, with the value at 0 d set to 1. The materials were collected and treatments conducted as described by Xu *et al.* (2014). Error bars indicate SE from three biological replicates. LSD indicates least-significant difference at *P*=0.05.

HT and LTC treatments both alleviate chilling-induced loquat fruit lignification to some extent (Xu *et al.*, 2014; Zeng *et al.*, 2015). Thus, the relationship between NAC genes and fruit lignification was investigated using fruit subject to HT and LTC (Xu *et al.*, 2014). mRNAs for *EjNAC3* and *EjNAC4* accumulated in response to 0 °C, and at 8 d showed 38-fold and 22-fold increases, respectively (Fig. 2). The transcript levels for each gene were significantly depressed by prior HT or LTC. For example, the relative level of *EjNAC3* at 4 d was 5.5 and 9.7 in HT and LTC fruit, compared with 25.1 for control fruit (0 °C), and this difference was even more marked at day 8 (Fig. 2). *EjNAC4* transcripts also increased, but this was initiated two days later, and although prior HT and LTC reduced transcript levels, HT was less effective than LTC (Fig. 2).

### Trans-activation by NAC genes of promoters of genes involved in lignin biosynthesis

**Fig. 3.**
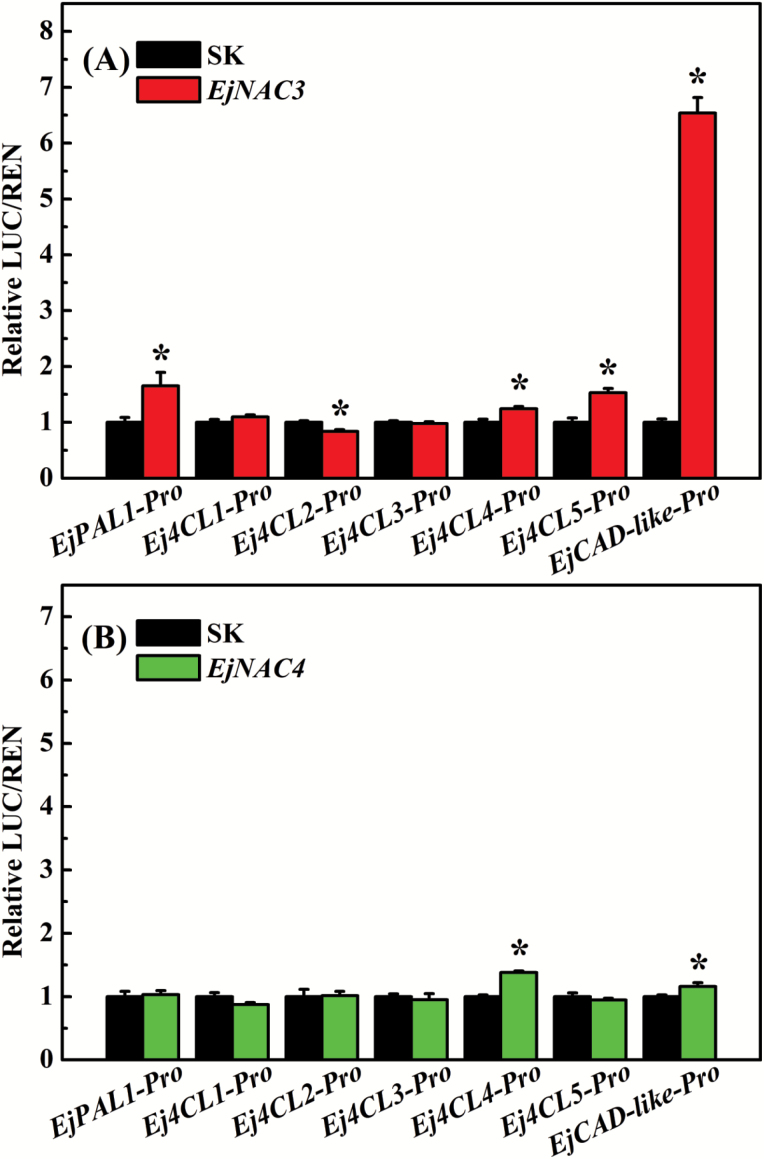
Regulatory effects of (A) EjNAC3 and (B) EjNAC4 on the promoters of loquat lignin biosynthesis genes as determined using dual-luciferase assays. The ratio of LUC/REN fluorescence obtained with the empty vector (SK) plus the promoter was used as a calibrator (set as 1). Error bars indicate SE from five replicates. Asterisks indicate that the difference between the control and treatment is significant at *P*<0.05.

According to data derived from real-time PCR, *EjNAC3* and *EjNAC4* transcript levels are highly correlated with lignification of fruit flesh (Xu *et al.*, 2014), which implies that these NAC genes might act as transcriptional regulators of lignin biosynthesis genes. To verify this conjecture, previously isolated promoters of seven genes from the phenylpropanoid pathway, namely *EjPAL1*, *Ej4CL1*, *Ej4CL2*, *Ej4CL3*, *Ej4CL4*, *Ej4CL5*, and *EjCAD1*, were selected for testing. Of these, *Ej4CL1* and *EjCAD1* were considered to be particularly important because they had been previously implicated in the control of lignification (Shan *et al.*, 2008; Xu *et al.*, 2014). Isolation of the full-length *EjCAD1* indicated that it is an atypical CAD member, with only the NAD(P)-binding Rossmann-fold domain, and lacking the conserved zinc-binding signature GHEXXGXXXXXGXXV (Kim *et al.*, 2004). Therefore, it was renamed as *EjCAD-like* (Genbank no. EF685346). Subcellular analysis indicated that *EjNAC3* was localized in the nucleus (see Supplementary Fig. S1), which is similar to most transcription factors. The dual-luciferase assay indicated that promoter activity of *EjCAD-like* was increased 6.54-fold higher than the control after transient overexpression of *EjNAC3* in tobacco leaves, but *EjNAC3* had little effect on the other gene promoters tested (Fig. 3A). Despite the correlation of *EjNAC4* with fruit lignification (Fig. 2), it had very limited effects on the promoters of lignin biosynthesis genes (Fig. 3B).

### Interaction between EjNAC3 and the *EjCAD-like* promoter

**Fig. 4.**
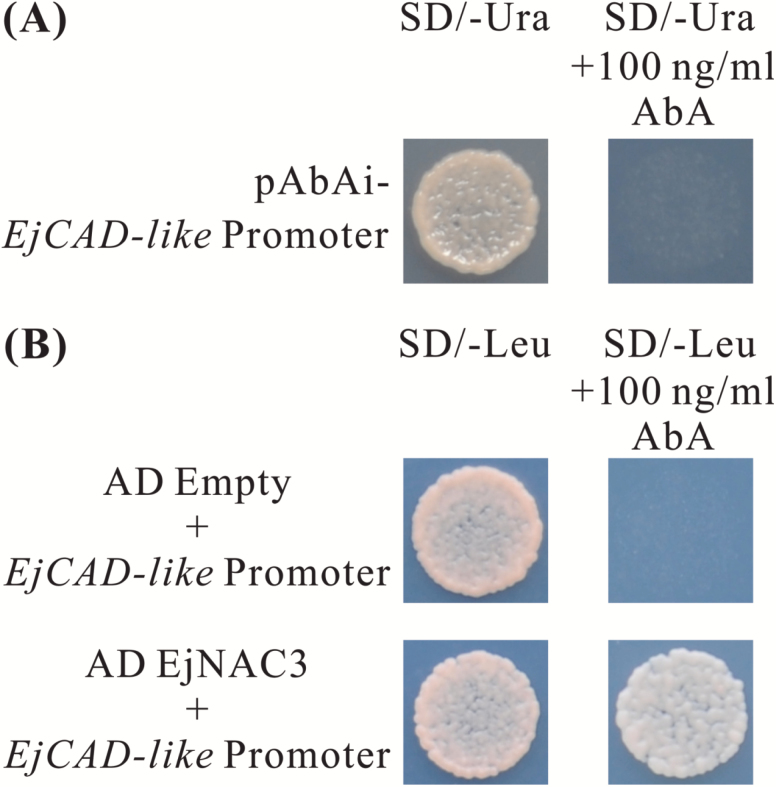
Yeast one-hybrid analysis of the ability of EjNAC3 to bind the promoter of *EjCAD-like*. (A) Autoactivation was tested on SD medium lacking Ura with 100 ng ml^–1^ Aureobasidin A (AbA). (B) Interactions were determined on SD medium lacking Leu in the presence of AbA (SD/–Leu +100 ng ml^–1^ AbA). The empty pGADT7 vector was used as a negative control.

**Fig. 5.**
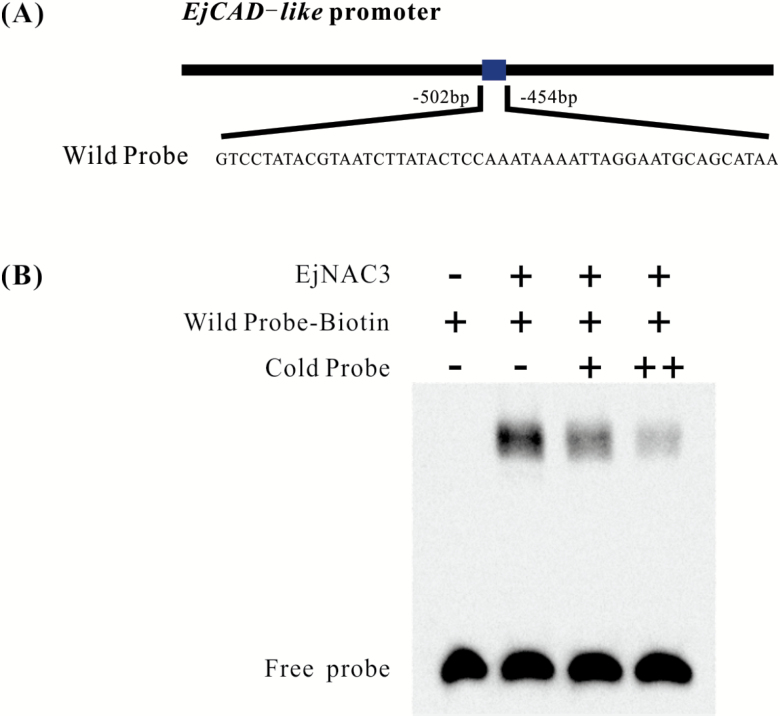
Electrophoretic mobility shift assay (EMSA) of EjNAC3 binding to the *EjCAD-like* promoter. (A) The probe sequence used for EMSA. (B) Purified EjNAC3 protein and biotin-labeled DNA probe were mixed and analysed on 6% native polyacrylamide gels. The presence (+) or absence (–) of specific probes is indicated. The concentration of the cold probe was 100 nM (+) or 300 nM (++), while that of the biotinylated probe was 1 nM.

In order to clarify the interaction between EjNAC3 and the *EjCAD-like* promoter, yeast one-hybrid assays were performed. The promoter sequence of *EjCAD-like* was inserted into the pAbAi vector and transformed into the Y1H strain. The results indicated that the *EjCAD-like* promoter was not activated without protein binding (Fig. 4A), and the interaction between EjNAC3 and the *EjCAD-like* promoter was therefore analysed. Yeast containing *EjNAC3* exhibited normal growth under 100 ng ml^–1^ AbA, while growth of the negative control with the empty vector was inhibited (Fig. 4B). EMSAs were performed aimed at locating more precisely the *cis*-element involved in binding. It has been reported that some NAC genes bind to TTGCGT or TTACGT core motifs (Lindemose *et al.*, 2014), and after searching the promoter of *EjCAD-like* we found there are five TTRCGT sites within an 870-bp region upstream of the start codon (Fig. 5A). Sequences from the region –502 bp to –454 bp were used as the native promoter. The biotinylated probe was able to bind EjNAC3 protein, and the addition of a high concentration of cold probe significantly reduced the binding affinity of the biotinylated probe. (Fig. 5B).

## Discussion

Lignin participates in various physiological processes, including the development of morphological characteristics, and the development of xylem strongly influences the conversion efficiency of lignocellulosic biomass to ethanol and the digestibility of forage crops (Jung, 1989; Ragauskas *et al.*, 2006; Fu *et al.*, 2011). Despite extensive studies on energy plants and model plants (e.g. *Populus*, *Eucalyptus*, switchgrass, sugarcane, and Arabidopsis), the regulatory mechanisms of fruit lignification are not well understood, and no transcription factors related to lignification have been reported in other fruit apart from loquat. Lignification in fruit is widely associated with stresses, such as chilling (Cai *et al.*, 2006*d*) and wounding (Kamdee *et al.*, 2014), and significantly influences fruit quality, consumer preference, and marketability. Thus, understanding the regulation of lignin biosynthesis is important not only in energy plants and model plant systems, but also for the fruit industry. Loquat fruit undergoes lignification after harvest, and LTC, HT, and application of MeJA have been developed to alleviate this (Cai *et al.*, 2006d;Cao *et al.*, 2009; Rui *et al.*, 2010). Moreover, the molecular basis for fruit lignification has also been investigated, and the role of some structural genes of the phenylpropanoid pathway has been reported (Shan *et al.*, 2008). In addition, four transcription factors, *EjMYB1*, *EjMYB2*, *EjAP2-1*, and *EjNAC1*, have been reported to be involved in lignification in loquat fruit. Further studies showed that the regulatory mechanisms of *EjMYB1* and *EjMYB2* were similar to their homologs from model plants (Xu *et al.*, 2014), while *EjAP2-1* was a novel regulator of lignin biosynthesis (Zeng *et al.*, 2015), and *EjNAC1* is an indirect regulator, although the underlying mechanisms have remained unclear (Xu *et al.*, 2015). Compared to the extensively reported transcription factors from energy plants and model species, and in view of its economic importance, the transcription factors involved in fruit lignification require more detailed investigation.

### Association between *EjNAC* expression and lignification of loquat fruit

In Arabidopsis, the NAC genes *AtNST1* and *AtNST2*, *AtVND1* to *AtVND7*, and *AtSND1* are top-layer regulators and have a strong influence on the synthesis of constituents of the secondary cell wall (Mitsuda *et al.*, 2005; Zhong *et al.*, 2006; Zhou *et al.*, 2014). Among these NAC genes, *AtVND*s are expressed specifically in vessels while *AtSND1*, *AtNST1*, and *AtNST2* are expressed in xylem fibers and extraxylary fibers or the anther endothecium. Mutation of these important regulatory genes results in abnormal secondary cell wall formation (Mitsuda *et al.*, 2005; Zhong *et al.*, 2006). Loquat *EjNAC1* is associated with lignin accumulation, and is capable of trans-activation of the promoters of *EjPAL1* and *Ej4CL1*. However, *EjNAC1* lacks a physical interaction with the target promoters (Xu *et al.*, 2015). In the current study, two new NAC genes were isolated, in addition to the previously reported *EjNAC1* and *EjNAC2*. The expression analysis indicated that *EjNAC3* and *EjNAC4* transcripts were positively correlated with lignin accumulation in fruit flesh in response to storage at 0 °C, and expression was inhibited by LTC and HT, with a corresponding alleviation of lignification (Xu *et al.*, 2014).

### *EjNAC3*, unlike most other lignin-related NAC genes, is a direct regulator of the lignin biosynthesis pathway

In Arabidopsis, NAC genes have mainly been considered as switches for secondary cell wall metabolisms, rather than being the direct regulators of structural genes (Zhong *et al.*, 2006), although there are rare exceptions such as *AtVNT7* and *AtSND1* (Zhao *et al.*, 2010; Yamaguchi *et al.*, 2011). However, enzymes of the phenylpropanoid pathway are not targets of *AtVNT7*, while *AtSND1* binds *Medicago truncatula MtF5H* but not the Arabidopsis endogenous homolog. The present findings using dual-luciferase assays, yeast one-hybrid assays, and EMSAs indicated that *EjNAC3* is a direct regulator of an *EjCAD-like* gene, via physical binding to TTRCGT motifs in the promoter. However, *EjNAC4* had very limited effects on lignin biosynthesis genes. Further investigation of the relationship between *EjNAC3* and known loquat fruit lignification-related transcription factors showed that both EjNAC3 and EjNAC4 had little effect on the promoters of *EjMYB1*, *EjMYB2*, and *EjAP2-1* (data not shown). In addition, *EjNAC3* is not only structurally different from most master-switch NAC members, but also NACs such as *AtVND7* and *AtSND1* that are capable of binding promoters of *AtCesA4* and *MtF5H*, respectively (Zhao *et al.*, 2010; Yamaguchi *et al.*, 2011). Taken together, this evidence indicates that *EjNAC3* is a new NAC transcription factor with a distinct role in regulating lignin biosynthesis.

The second candidate gene, *EjNAC4*, with a similar expression pattern to *EjNAC3*, was not, however, able to influence the activities of the target-gene promoters tested. It is possible that *EjNAC4* might play a similar role to its homolog *AtSND2*, whose target is *AtMYB103* (Hussey *et al.*, 2011), and this is something that should be tested.

### Role of EjNAC3 in fruit-flesh lignification

**Fig. 6.**
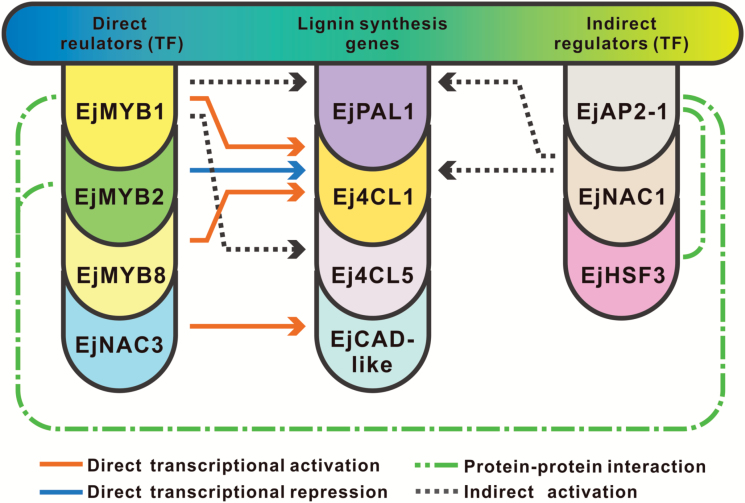
The regulatory network of lignin biosynthesis in loquat fruit. Although the network that regulates key enzymes in the phenylpropanoid pathway is far from complete, some transcription factors together with their targets have been identified, including NAC-domain proteins, MYB-domain proteins, a HSF protein, and an AP2-domain protein. Generally, these transcription factors can be classified into two groups: direct regulators that have physical interactions with identified target promoters, and indirect regulators that activate or repress expression of specific genes via pathways yet to be established.

According to earlier studies (Xu *et al.*, 2014; Wang *et al.*, 2016), in loquat, *Ej4CL1* was the only enzyme to be affected by previously characterized transcription factors, such as *EjMYB1*, *EjMYB2*, and *EjMYB8* (Fig. 6). The lignin biosynthesis pathway is composed of 11 enzymes in addition to 4CL (Boerjan *et al.*, 2003). Theoretically, each step in the pathway could be accessible to regulators. The discovery of *EjNAC3* identifies a new regulatory step in the pathway, in addition to the control of 4CL. CADs catalyse the last step of monolignol synthesis, and the interaction between *EjNAC3* and *EjCAD-like* broadens the regulatory range of lignin-related transcription factors. So far, two NAC family genes have been confirmed as being involved in fruit lignification, *EjNAC1* (Xu *et al.*, 2015), which regulates *EjPAL1* and *Ej4CL1*, and *EjNAC3*. Interestingly, they seemed to function in parallel, since their targets are different. These clues indicate the complicated roles that NAC genes play in the hierarchy of regulatory interactions in the overall network (Fig. 6).

In the present study, a chilling-inducible NAC transcription factor (*EjNAC3*) was characterized from loquat fruit. Phylogenetic analysis of its amino acid sequence indicated that it has a distinct structure compared with SND, NST, and VND genes. Dual-luciferase assays, Y1H assays, and EMSAs suggested that EjNAC3 could recognize and regulate the promoter of an *EjCAD-like* gene, indicating that EjNAC3 has the ability to directly regulate structural genes, which only few NAC genes possess. In loquat, chilling, as an environmental stimulus, can increase lignin production in the fruit flesh by inducing transcripts of *EjNAC3* that act on a CAD-like gene.

## Supplementary Data

Supplementary data are available at *JXB* online.

Table S1. Primers for used for RACE.

Table S2. Primers used for real time PCR.

Table S3. Primers used for full-length sequences of EjNAC gene isolation.

Table S4. Primers used for pGADT-7 vector construction.

Table S5. Probes used for EMSAs.

Fig. S1. Subcellular localization of *EjNAC3*.

## Supplementary Material

supplementary_table_S1_S5

supplementary_figure_S1

## Abbreviations:

1-MCP: 1-methylcyclopropene
4CL: 4-coumarate-CoA ligase
AbA: aureobasidin A
ASA: acetylsalicylic acid
CAD: cinnamyl alcohol dehydrogenase
F5H: ferulate-5-hydroxylase
HT: heat treatment
LTC: low-temperature conditioning
PAL: phenylalanine ammonia lyase
