# Supplementary material for: EjNAC3 transcriptionally regulates chilling-induced lignification of loquat fruit via physical interaction with an atypical CAD-like gene
Source: J Exp Bot. 2017 Sep 16;68(18):5129–36. doi: 10.1093/jxb/erx330 (PMC5853329; doi:10.1093/jxb/erx330)
Supplement: supplementary_table_S1_S5 [file erx330_suppl_supplementary_table_s1_s5.pdf]

Appendix

Table S1 Primers for RACE

Gene		Primary primer (5' to 3')	Nested primer (5' to 3')
EjNAC3	5RACE	TGCCCGGTTTGTTCTCAGACCAGT	CAAGGCAAATCCCAAGGCTCACAC
	3RACE	CTTCCTCCGGGATTCCGATTTCATC	AGTGTGAGCCTTGGGATTTGCCTTG
EjNAC4	5RACE	ACGAAGACCCGTCACCTTGGACAAC	GACCTTTGAGACCACCAGCTCACCA
	3RACE	CATGCACCAATACCACCTTGGCAAC	GGGCGACAATAGAGAAAGCCCACCT

Table S2 Primers for Realtime PCR

Gene	Forward Primer (5' to 3')	Reverse Primer (5' to 3')
EjNAC3	GTACTCGTCCGCTCCGATAG	GCACTCCCTTGGTTCTTCAA
EjNAC4	TGTGAATATAAGAGGTGGGCAAG	GAAAGGACCTCCAGGATCATAGT

Table S3 Primers for full length sequences of *EjNAC* genes isolation

Gene	Forward Primer (5' to 3')	Reverse Primer (5' to 3')
EjNAC3	ATGGAAAATACTTCAGGGTTTA	TCAATAATTCCAGAGGCCATCGA
EjNAC4	ATGACATGGTGCAATGACTCCGA	CTATTTCCTCTCAAGCTTCCCTT

Table S4 Primers for pGADT-7 vector construction

Gene	Forward Primer (5' to 3')	Reverse Primer (5' to 3')
EjNAC3	GAATTCATGGAAAATACTTCAGGGTT	GGATCCTCAATAATTCCAGAGGCCA

Table S5 Probes used for EMSA assay

**Biotinylated wild probe F**	GTCCTATACGTAATCTTATACTCCAAATAAAATTAGGAATGCAGCATAA
**Biotinylated wild probe R**	TTATGCTGCATTCCTAATTTTATTTGGAGTATAAGATTACGTATAGGAC
