# Supplementary material for: EjNAC3 transcriptionally regulates chilling-induced lignification of loquat fruit via physical interaction with an atypical CAD-like gene
Source: J Exp Bot. 2017 Sep 16;68(18):5129–36. doi: 10.1093/jxb/erx330 (PMC5853329; doi:10.1093/jxb/erx330)

Fig. S1 Localization of EjNAC3. Vector contain mCherry (35S::mCherry) was used indicating location of nucleus. EjNAC3 fused with GFP was detected only in the nucleus.

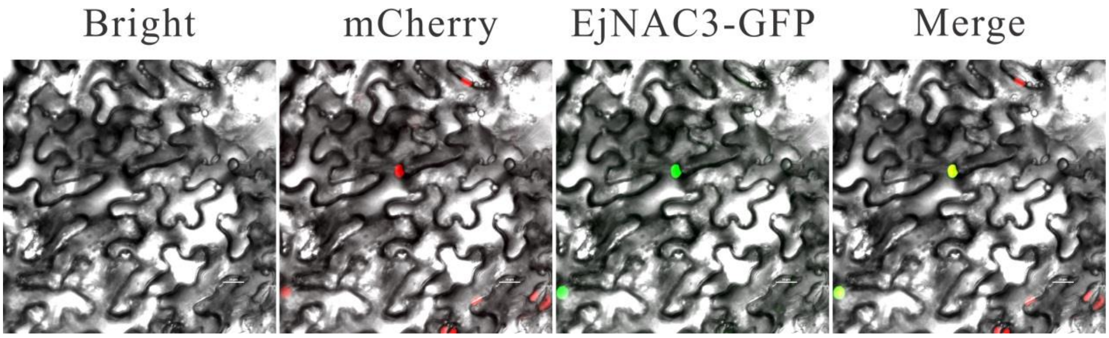

Supplement: supplementary_figure_S1 [file erx330_suppl_supplementary_figure_s1.pdf]
